# How to compare antivirals in the treatment of chronic hepatitis B?

**DOI:** 10.1186/1476-0711-8-6

**Published:** 2009-03-02

**Authors:** Resat Ozaras, Hakan Leblebicioglu

**Affiliations:** 1 Istanbul University, Cerrahpasa Medical School, Department of Infectious Diseases and Clinical Microbiology, Istanbul, Turkey; 2 Ondokuz University, Medical School, Department of Infectious Diseases and Clinical Microbiology, Samsun, Turkey

## Editorial

After the use of antiviral drugs in the treatment of hepatitis B virus (HBV) infection, we found new therapeutic options for non-responders to interferon (IFN). Additionally, those could not be treated previously by IFN, such as cirrhotics have been given effective antivirals. However, the parameters to determine the efficacy, the outcome measures, and the duration of the treatment emerged as unresolved issues by the treatment with antivirals. Resistance to antivirals is also a challenge and its definition, detection, and management are new controversial topics in the area of HBV treatment.

Now, the clinician has a wider choice of antivirals: in addition to IFNs, lamivudine, adefovir, entecavir, telbivudine, and tenofovir have been approved for treating chronic HBV infection [1,2]. As the HBV treatment studies are published, we see several comparisons of the antivirals [3-5]. Updated EASL guideline gave the comparisons of the HBV drugs [6]. However comparing efficacies or other aspects of two or more antivirals in a simple way may give misleading results.

Recently, Feld and Ghany [7] summarized the complexity of the issue of hepatitis B treatment and simply compared the response to approved antiviral agents among hepatitis B e antigen-positive patients. The reviewed studies are the main ones showing the efficacy of the given drug and mostly represent the ones submitted to FDA. They generally include sufficient number of patients to conclude about the efficacy of the drugs. Lamivudine was the comparator in some of the studies and the results obtained from the use of lamivudine were reported as well. There are several differences between these studies by many aspects (Table 1) [8-12]. Different methods were used in these studies for detection and quantification HBV DNA. Also sensitivity, detection limit and dynamic range of quantification of these tests make them difficult to compare [13]. Only baseline HBV level which is lower more than 1 log (corresponds to several folds) in adefovir study, for example, makes it almost impossible to compare. Combined data of three studies were given for lamivudine, however virological response criterion differed among these studies.

**Table 1:** Differing characteristics of the studies comparing the response rates in HBeAg-positive patients

	**Pegylated-IFN-α2a, 180 μg once Weekly** [8]	**Lamivudine 100 mg/d** [8-10]	**Adefovir 10 mg/d** [11]	**Entecavir 0.5 mg/d** [9]	**Telbivudine 600 mg/d** [10]	**Tenofovir 300 mg/d** [12]
Mean age (years)	32.5	31.6 to 35	34	35	32	34
Male gender (%)	79	74 to 79	76	77	74	68
Asian race (%)	87	57 to 87	60	58	82	36
Patient number	271	272 to 463	171	354	399	176
Previous treatment	Conventional IFN 11%, lamivudine 11%	Conventional IFN 11%/IFN 13% and lamivudine 3%/NA	IFN (24% among all arms)	IFN 13% and lamivudine 3%	NA	IFN 17%, lamivudine or emtricitabine 5%
Baseline HBV-DNA (log_10 _cp/ml)	9.9	9.5 to 10.1	8.25	9.62	9.51	8.64
Baseline ALT	114.6	102 to 159	139	140.5	146	142
Baseline necro-inflammatory score	NA	7.3 to 7.7	7.37	7.8	7	8.3
Baseline fibrosis score	NA	2.2 to 2.3	1.64	2.3	2.2	2.3
Genotypes (%, A/B/C/D/others)	8/28/60/3/1	7/30/58/4/1 (ref 8) 28/22/25/15/11 (ref 9)	NA	27/19/31/10/13	NA	24/14/25/32/5
Treatment duration (weeks)	48	48 to 52	48	48	52	48
Virological response criterion (HBV DNA level)	<400 cp/ml	<400 cp/ml (ref 8), <0.7 MEq/ml (ref 10), <300 cp/ml (ref 10)	<300 cp/ml	<0.7 MEq/ml	<300 cp/ml	<400 cp/ml

IFN: interferon, NA: not available, MEq/ml: mili-equivalent/mililiter, cp/ml: copies/milliliter.

ALT is another issue: Baseline mean ALT levels may not be comparable. Additionally, ALT level as an inclusion criterion varied: greater than 1 time the upper limit of the normal range for pegylated interferon [8], 1.3 times for entecavir [9] and telbivudine [10], and 1.2 times for adefovir [11].

Patient numbers, genotype distributions, treatment durations, previous treatment, and probably many other differences exist among the studies compared, discouraging to make a head-to-head comparison. Asian race for example, is associated with poor response to interferon treatment [14], and the rate of this race is highest in pegylated IFN study.

Considering the role of high ALT and low DNA on treatment response, a comparison of efficacy of any two drugs should include the details of distribution of these parameters in the study population. Only the telbivudine study described the stratification of the patients according to DNA levels.

Resistance is another important issue in the treatment of HBV with antivirals. The published clinical trials have used varying definitions of efficacy, failure, and resistance based on different measures of virologic responses [15]. If we analyze the resistance outcomes in the studies, we see that although some studies included all patients to the tests for antiviral resistance [16,17] some others included only those with a viral rebound [18,19]. The methods used to detect the resistance also differ: Although the majority of the studies used direct sequencing [20], restriction fragment length polymorphism [21] and line-probe, matrix-assisted laser desorption/ionization time-of-flight mass spectrometry [22] were used in some others. Study design differs in the studies and reported resistance rates should be evaluated regarding the design. In entecavir study, patients who had a response (defined by an HBV DNA level below 0.7 megaequivalents [MEq] per milliliter and HBeAg loss) or a nonresponse (defined by an HBV DNA level of 0.7 MEq per milliliter or greater) discontinued study treatment at week 52 [9]. Patients who had only a virologic response (defined by an HBV DNA level below 0.7 MEq per milliliter and no HBeAg loss) were offered continued study therapy for up to 96 weeks. This design, especially discontinuation of the therapy in nonresponders may decrease the risk of resistance. This unique feature of the study may underestimate the resistance.

Studies in HBeAg-negative patients may have similar problems, especially in inclusion criteria and response definitions [23]. A review defined virological response as "decrease of serum HBV DNA to PCR-undetectable levels (preferably) or <2000 IU/mL (4 log10 copies/mL) [5]. The entecavir study used "HBV DNA <300 copies/ml by PCR assay" [24] and the adefovir study used "<400 copies/ml" [25]. Clevudine study, finally, used hybride capture with lower limit of detection of <4700 copies/mL and then if it remains undetectable, used PCR with lower limit of detection of 300 copies/mL [26].

A simple comparison of several drugs in differing study characteristics may not give reliable conclusive information to the reader. Unless randomizing the patients in the same study design, it seems difficult to compare the efficacies of any given drugs. When giving such comparisons, the reader should be warned against the difference among study characteristics and better, the main differences should also be included.

## Competing interests

The authors declare that they have no competing interests.
